# Notes and Notices

**Published:** 1893-12

**Authors:** 


### NOTES AND NOTICES.

Mr. W. B. Saunders, Publisher, 925 Walnut St., Philadelphia, Pa., writes: "We now have the entire manuscript of the second volume of Dr. Pepper's *Theory and Practice of Medicine* in our printer's hands, and we can assure the profession at large that the work will be placed in their hands within the next six weeks. There is one feature about this book to which I would call your special attention. In the first volume nearly two hundred pages are from the pen of Dr. William Pepper, and in the second volume he writes over three hundred pages, thus making over one-fourth of the entire work from the Editor."

Dr. Arthur G. Allan, the homœopathic eye specialist, has associated himself with Dr. Thomas M. Dillingham, at 46 West Thirty-sixth Street, New York City.

Reed & Carnick's Infant Food.—The highest awards and medals at the World's Fair were given to *Reed & Carnick's Infant Foods and Kumysgen.*

Mr. W. B. Saunders, Publisher, of Philadelphia, writes: "We will send you by mail in a few days a copy of *Senn's Syllabus of the American Text-Book of Surgery*, which we think will be a valuable aid to all who now possess the *American Surgery.* This latter book, as I have written you before, has had a phenomenal sale, over ten thousand copies having been sold last year."

